# A case of failed awake craniotomy due to progressive intraoperative hyponatremia

**DOI:** 10.1186/s40981-018-0176-z

**Published:** 2018-05-15

**Authors:** Suguru Yamamoto, Hanayo Masaki, Kotoe Kamata, Minoru Nomura, Makoto Ozaki

**Affiliations:** Department of Anesthesiology, Tokyo Women’s Medical University, 8-1 Kawada-cho, Shinjuku-ku, Tokyo, 162-8666 Japan

**Keywords:** Hyponatremia, Awake craniotomy, Mannitol, Carbamazepine

## Abstract

**Background:**

Perioperative seizure control is correlated with a better surgical outcome for awake craniotomy, but some anticonvulsants can induce hyponatremia. Mannitol has also been reported to be hyponatremic.

**Case presentation:**

A 51-year-old right-handed man had malignant glioma in the left parietal lobe. Since anticonvulsant polytherapy did not stop his seizure activity, the daily dose of carbamazepine was increased beginning 17 days before awake craniotomy. The last preoperative blood examination indicated that his plasma sodium level had gradually decreased from 140 to 130 mEq/L. Following skin incision, 200 mL of 20% mannitol was administered and his plasma sodium level subsequently dropped to 117 mEq/L. The surgical strategy was changed so that the entire procedure would be performed under general anesthesia because severe intraoperative complications were anticipated.

**Conclusions:**

This case suggests that a perioperative electrolyte imbalance caused by drug interactions could be clinically significant for awake craniotomy.

## Background

Hyponatremia is a common disorder that is frequently iatrogenic [1]. In patients with mild chronic hyponatremia, suitably sensitive tests reveal instability in dynamic tasks and attentional impairment [2]. While mannitol is given to neurosurgery patients to reduce intracranial pressure (ICP) [3, 4], the optimal conditions of treatment are unclear [5]. The effect of mannitol on ICP is dose-dependent, and its rapid infusion can lead to an electrolyte imbalance [6]. In awake craniotomy, surgery-related complications contribute to surgical and functional outcomes [7]. Intraoperative seizure complicates surgery by affecting functional mapping and the patient’s level of consciousness. Therefore, careful preoperative loading of anticonvulsants is recommended [8].

In this case, planned awake craniotomy was modified due to progressive hyponatremia.

## Case presentation

### Preoperative course

A 169-cm, 65-kg, 51-year-old, right-handed man with right-side hemiparesis and dysarthria underwent screening magnetic resonance imaging (MRI) 2 years before craniotomy: a diffuse parietal tumor was misdiagnosed as cerebral infarction. Anticonvulsant polytherapy (carbamazepine, 200 mg daily; levetiracetam, 1000 mg daily) was started for symptomatic epilepsy. Right-hand and oro-facial motor paresis worsened beginning 4 months before surgery, and he was diagnosed with malignant glioma. He was referred to our institution 2 months before surgery. MRI demonstrated a T1 hypointense, T2/fluid-attenuated inversion recovery hyperintense area in the left lower parietal lobe with a gadolinium-positive mass in the postcentral gyrus. ^11^C-methionine positron emission tomography showed a high accumulation of methionine mainly in the postcentral gyrus. The results of a manual muscle test (MMT) showed reduced function (3/5) in his right-upper extremity.

The patient’s hypertension was well-controlled with telmisartan (40 mg, daily) and hydrochlorothiazide (12.5 mg, daily). Diabetes was treated with glimepiride (1 mg, daily) and anagliptin (400 mg, daily). Obstructive sleep apnea syndrome (OSAS) was suspected due to a history of snoring. Electrolyte imbalance was not observed. Although carbamazepine was increased to 400 mg daily beginning 17 days before surgery, epileptic seizure in his right-upper limb occurred every 2–3 days. Levetiracetam was increased to 2000 mg daily upon admission 4 days before surgery.

The lesion, suspected grade III glioma according to the World Health Organization (WHO) guidelines [9], was distributed throughout the primary motor and sensory areas of his dominant hemisphere. We chose awake craniotomy using the “Asleep-Awake-Asleep” technique combined with intraoperative MRI for removal of his glioma. Uncontrollable seizure suggested rapid growth of the tumor. The patient was evaluated as American Society of Anesthesiologists (ASA) Physical Status Class 2. While the preoperative blood examination at admission indicated that his plasma sodium level (reference 136–145 mEq/L) had decreased from 140 to 130 mEq/L, he was asymptomatic. Preoperative correction of hyponatremia was ruled out to avoid neurological deterioration due to further growth of the glioma. Additional blood or urine examinations were not performed.

### Awake craniotomy

No premedication was given. Besides standard ASA monitoring, invasive arterial pressure and processed electroencephalogram monitoring were adopted. Following the intravenous administration of 100 μg fentanyl, general anesthesia was commenced and maintained with the target-controlled infusion of propofol (4.0 μg/mL) and the continuous infusion of remifentanil (0.5 μg/kg/min). After several attempts, a supraglottic airway (SGA) was inserted without muscle relaxant. Ventilation was adjusted to maintain an EtCO_2_ level around 35 mmHg. Normal saline and bicarbonate Ringer’s solution were given. Scalp blocks and infiltration analgesia were provided with 60 mL of 0.3% ropivacaine and 20 mL of 1% lidocaine with 0.01% epinephrine, respectively. Arterial blood gas (ABG) analysis (STAT PROFILE® Critical Care Express; Nova Biomedical, Waltham, MA) after induction showed mild hyponatremia (Table 1, T1).

**Table 1:** Results of arterial blood gas analysis

		T1	T2	T3	T4	T5	T6	T7
pH		7.397	7.392	7.401	7.403	7.415	7.431	7.440
PaO_2_	[mmHg]	214.2	218.6	218.7	220.9	222.0	226.3	220.7
PaCO_2_	[mmHg]	35.6	36.2	35.3	37.4	35.5	31.3	32.3
HCO^3−^	[mmol/L]	22.2	22.3	22.2	23.6	23.0	20.9	22.1
Na^+^	[mEq/L]	124.5	117.8	120.1	121.7	123.9	125.6	124.9
K^+^	[mEq/L]	4.30	5.58	5.82	4.41	4.25	4.41	4.62
Glucose	[mg/dL]	136	168	169	273	185	160	157

T1 = after induction (38 min after induction); T2 = 25 min into mannitol infusion (117 min after induction); T3 = start glucose-insulin therapy (134 min after induction); T4 = after glucose-insulin therapy (191 min after induction); T5 = follow-up analysis (236 min after induction); T6 = follow-up analysis (313 min after induction); T7 = end of surgery (369 min after induction)

Hypotension followed by induction was stabilized by three boluses each of ephedrine 4 mg and phenylephrine 0.1 mg until the beginning of surgery. Before dural incision, 200 mL of 20% mannitol was administered over 10 min. ABG analysis 117 min after induction, 25 min into mannitol infusion, revealed hyponatremia with hyperkalemia (Table 1, T2). Neither pathological electrocardiogram changes nor arrhythmia was observed. Sodium bicarbonate 2.8 g was given. Glucose-insulin (40 mL of 50% glucose containing 8 units of insulin) was infused simultaneously with another ABG analysis, which was performed to exclude mechanical failure (Table 1, T3). Hyponatremia was not treated promptly because of the risk of central pontine myelinolysis. ABG analysis 191 min from induction indicated persistent hyponatremia without hyperkalemia (Table 1, T4). In the planned transition to the awake phase, we anticipated insufficient wakefulness, intractable seizure, and deterioration of upper airway patency. Thus, the entire procedure was performed under general anesthesia. The remaining operation was uneventful, although the plasma sodium level remained less than 125 mEq/L (Table 1, T5–7). Anesthetics were discontinued, and SGA was removed 415 min after induction. The patient’s cardiorespiratory status was stable, but the patient could not follow orders. An intravenous transfusion of 1500 mL bicarbonate Ringer’s solution, 300 mL 1% glucose-containing acetated Ringer’s solution, 650 mL normal saline, and 200 mL mannitol was given. Total urine output was 1300 mL, and estimated blood loss was 36 mL. Surgery and anesthesia lasted 433 and 354 min, respectively.

### Postoperative course

The patient regained consciousness 1 h after the operation. MMTs of his right upper and lower extremities showed 1/5 and 3/5, respectively. Motor aphasia persisted until eight postoperative days (POD), and he walked unaided on POD 24. His plasma sodium concentration normalized by POD 35, before discharge on POD 39. MMTs at discharge were 2/5 and 4/5, respectively. Since the pathological examination revealed an oligoastrocytoma (WHO grade II), he received adjuvant chemotherapy. Prophylactic anticonvulsant polytherapy was continued, and there was no evidence of tumor recurrence at 36 months follow-up.

## Discussion

Though awake craniotomy possibly gives better outcomes than that under general anesthesia, the plasma sodium level must be carefully monitored, because awake craniotomy cannot be achieved if the patient shows neurological sequelae during awake phase.

While mild hyponatremia (plasma sodium 130–135 mEq/L) is generally regarded as asymptomatic, gait disturbance and attentional impairment are often seen [2]. When plasma sodium falls below 125–130 mEq/L, the patient may complain of nausea or malaise. Headache, lethargy, restlessness, and disorientation may occur at 115–120 mEq/L. More severe and rapidly evolving hyponatremia can lead to seizure, coma, or even respiratory arrest [10]. However, aggressive correction of the plasma sodium concentration is not recommended, since it also correlates with neurological sequelae [10]. We were concerned that carbamazepine along with hydrochlorothiazide could cause syndrome of inappropriate antidiuretic hormone secretion (SIADH), though a definitive diagnosis was not obtained due to incomplete laboratory examinations [1, 11]. Also, intraoperative mannitol might have promoted hypertonic hyponatremia by driving fluid from the intracellular to the extracellular space. This case suggests that a plasma sodium disorder must be compensated before surgery; the possibility of hyponatremia-induced neurological symptoms prevented us from switching to the awake phase. Respiratory exacerbation was also anticipated in our OSAS-complicated patient. Intraoperative seizure should be controlled due to the risk of physical harm to the patient. Moreover, Todd paralysis or postictal aphasia due to seizure will preclude awake craniotomy. Therefore, in the Japanese guidelines for awake craniotomy, the anticonvulsant level preceding surgery should be maintained within a therapeutic range [8]. In a retrospective analysis of 477 awake craniotomy cases, preoperative seizure and anticonvulsant polytherapy were related to surgical failure due to intraoperative seizure [7]. Furthermore, patients who experienced seizure during awake craniotomy had a higher incidence of short-term motor deterioration and a longer hospitalization [12]. While anticonvulsant polytherapy is a common strategy for intractable seizure, carbamazepine, valproate, lamotrigine, and oxcarbazepine cause SIADH [13]. Severe hyponatremia is uncommon with a single agent [11]. Preoperative seizure control is important for successful awake craniotomy, and a preoperative electrolyte imbalance, especially hyponatremia, should be treated as much as possible.

Rapid infusion of mannitol increased plasma osmolality, which effected the transcellular redistribution of sodium and potassium. High-dose (2 g/kg) mannitol produced a significantly greater decrease in serum sodium than low-dose mannitol (1 g/kg) (− 20.7 vs. − 8.7 mEq/L, respectively) and a greater increase in serum osmolality [6]. Though these changes recovered to the preoperative level without correction when the patient returned to the recovery room, a serum sodium concentration that started to decline from the beginning of mannitol infusion remained below 130 mEq/L during craniotomy [5]. In our case, 0.4 g/kg mannitol was administered over 10 min, and the plasma sodium concentration was lowest (117 mEq/L) 15 min after infusion. In awake craniotomy, there is less time between the need for mannitol and patient awakening than with general anesthesia. Accordingly, hypertonic saline (HS) may be a better choice in awake craniotomy. In a recent meta-analysis that compared equiosmolar HS to mannitol during craniotomy under general anesthesia, HS gave better brain relaxation without a significant increase in urine volume [14]. Also, while 3% HS produced clinically acceptable hypernatremia for 4 h, equiosmolar 20% mannitol produced hyponatremia for 2 h [15]. Another clinical study under a standard depth of anesthesia and hemodynamic parameters showed similar results [16]. Thus, HS may replace mannitol due to its ability to increase osmolarity and decrease ICP while maintaining an appropriate serum sodium level for awake craniotomy.

## Conclusions

Plasma sodium affects neurological sequelae; if nausea, restlessness, disorientation, or seizure occurs in an awake patient, awake craniotomy might not be completed. The interactions of perioperative medications and electrolyte imbalances should be carefully monitored.

## Abbreviations

ABG: Arterial blood gas
ASA: American Society of Anesthesiologists
HS: Hypertonic saline
ICP: Intracranial pressure
MMT: Manual muscle test
MRI: Magnetic resonance imaging
OSAS: Obstructive sleep apnea syndrome
POD: Postoperative days
SGA: Supraglottic airway
SIADH: Syndrome of inappropriate antidiuretic hormone secretion
WHO: World Health Organization

## Authors’ information

SY is a senior resident in the Department of Anesthesiology at Tokyo Women’s Medical University. HM is a senior resident in the Department of Anesthesiology at Tokyo Women’s Medical University. KK is an assistant professor in the Department of Anesthesia at Tokyo Women’s Medical University. MN is a professor in the Department of Anesthesia at Tokyo Women’s Medical University. MO is a professor in the Department of Anesthesia at Tokyo Women’s Medical University.
